# Adaption and validation of the adherence barriers questionnaire for HIV patients on antiretroviral therapy (ABQ-HIV)

**DOI:** 10.1186/s12879-018-3530-x

**Published:** 2018-11-28

**Authors:** Sabrina Mueller, Thomas Wilke, Vanessa Gorasso, Marc Erhart, Jens M. Kittner

**Affiliations:** 1 Ingress-Health HWM GmbH, Wismar, Germany; 2 Universitaetmedizin Mainz, Universitaetsmedizin, Germany; 3 Klinikum Darmstadt, Universitaetsmedizin, Germany

**Keywords:** Adherence, Non-adherence, Adherence barriers, Human immunodeficiency virus (HIV), Antiretroviral therapy (ART), Adherence barriers questionnaire (ABQ), Patient questionnaire

## Abstract

**Background:**

Despite substantial advances in antiretroviral therapy (ART) for human immunodeficiency virus (HIV) in the last decades, non-adherence (NA) continues to be a major challenge in the real-life treatment. To meet this challenge, adherence-promoting interventions with a tailored approach towards patient-specific adherence barriers that are identified using a reliable and practicable questionnaire are needed. The aim of this investigation was to develop and validate a respective questionnaire (Adherence Barriers Questionnaire for HIV: ABQ-HIV), based on an earlier version of the ABQ.

**Methods:**

The existing ABQ was discussed by an expert panel and revised according to the specifications of ART therapy for HIV patients. Initially, the ABQ-HIV consisted of 17 items formulated as statements (4-point-Likert-scale ranging from “strongly agree” to “strongly disagree”). A higher score indicates a higher influence of a certain barrier on patient’s perceptions. The ABQ-HIV was applied in a cross-sectional survey of German HIV patients. Evaluation of the questionnaire included an assessment of internal consistency as well as factor analysis. Convergent validity was assessed by comparing the ABQ-HIV score with the degree of self-reported adherence measured by the 8-item Morisky Medication Adherence Scale (MMAS-8©).

**Results:**

Three hundred seventy patients were able to be included in all validation analyses. The included patients had a mean age of 51.2 years, and 15.7% were female. The mean HIV infection time was 11.7 years, and the mean duration of treatment since first starting ART was 8.7 years.

Twenty-five patients – excluded from all further analyses - were not able/willing to answer all ABQ-HIV questions.

The results of the reliability analysis showed a Cronbach’s α of 0.708 for the initial 17-items in the ABQ-HIV draft. Two items were eliminated from the initial questionnaire, resulting in a Cronbach’s α of 0.720 and a split-half reliability of 0.724 (Spearman–Brown coefficient).

Based on the reduced 15-item scale, the factor analysis resulted in three different components of the questionnaire. Component 1, with seven items, represents the unintentional adherence barriers. The second component, which contains five items, can be labelled as a subscale describing barriers associated with disease/treatment knowledge. Finally, three items, which can be summarized as intentional adherence barriers, show maximum loading in the third component.

The score of the reduced 15-item ABQ-HIV scale, as well as the scores of the three subscales, correlated significantly with the MMAS score. All correlation coefficients were negative, indicating that higher burdens of adherence barriers measured by ABQ-HIV or its subscales were associated with a lower MMAS score and thus, with a lower adherence level.

The ROC analysis using the MMAS low adherence classification as its state variable provided a cut-off for the ABQ-HIV scale of > 28 (sensitivity: 61.5%, specificity: 83.3%). In our sample, 85 patients (23.0%) reached a score of > 28 and appeared to face a high non-adherence risk.

**Conclusions:**

The ABQ-HIV is a practical, reliable, and valid instrument for identifying patient-specific barriers to adherence in the HIV treatment. It is also useful in identifying HIV patient subgroups, according to adherence barriers specific to these patients.

**Electronic supplementary material:**

The online version of this article (10.1186/s12879-018-3530-x) contains supplementary material, which is available to authorized users.

## Background

Medication adherence, which can be defined as the extent to which a patient’s medicine-taking behavior corresponds with agreed instructions from a health care provider [1–3], is essential for realizing the potential benefits of most medication-based treatment [1–4]. Many patients, especially those with chronic diseases, experience difficulties in adhering to a recommended treatment plan. Medication non-adherence (NA), with average rates of those affected being between 30 and 50%, is a major challenge in the real-life treatment of these patients [3, 4].

Despite several advances in antiretroviral therapy (ART) for human immunodeficiency virus (HIV) in the last decades [5], non-adherence (NA) continues to be a critical phenomenon in the treatment of these patients. Lacking adequate adherence, sufficient concentrations of antiretroviral agents to suppress HIV replication are not maintained in infected cells. Not only short-term virological response will be poor, but low drug concentrations also dramatically accelerate development of drug-resistance [6]. Therefore, strict adherence to ART is essential for treatment success [7, 8].

Several interventions to ensure and support adherence to ART have been tested, but results across several studies indicated a lack of efficacy so far [9]. One major reason may be the failure to successfully customize adherence interventions to patient-specific needs or preferences [3]: Existing research shows that the qualitative and quantitative contribution of a variety of different factors (adherence barriers) is vastly different for most patients [9].

In line with this, the World Health Organization (WHO) described NA as being a complex and multidimensional construct, which is related to socio-economic, health care system-related, disease- and therapy-specific as well as patient-related factors [10]. Therefore, recent research proposes to differentiate different types of NA, especially between intentional and unintentional NA [3, 11–13].

In order to differentially assess adherence barriers, a reliable and valid instrument to identify causes of NA in HIV patients is also needed. The “Adherence Barriers Questionnaire” (ABQ) has already been shown to be practical, reliable, and valid in chronic indications with self-administrated medication [14]. Therefore, the aim of the current study was to adapt and validate the ABQ to the specific needs of HIV patients.

## Methods

The previously developed ABQ was used as a basis. By reviewing the literature, it was found that no similar patient-report outcome measure addressing barriers of adherence in the implementation of ART therapy in HIV had been previously published. Initial adaptation of the ABQ was thoroughly discussed with an expert panel and was based on existing evidence on potential causes of NA in HIV patients. The panel included clinicians with several years of specific experience, as well as the developer of the original ABQ (first author of this manuscript). The ABQ research team assessed the suitability of the existing items and added additional items that were identified to be relevant in the HIV indication based on the results of the conducted literature review. Clinical experts were asked to evaluate the generated item collection with respect to their relevance and to add important features that could possibly affect patients’ adherence to medication which were not covered so far. After that, the drafted ABQ-HIV passed two revision rounds.

To evaluate the psychometric properties of the generated ABQ-HIV, a cross-sectional, non-interventional study of a cohort of HIV patients in southwest Germany was conducted. Nine outpatient specialists for infectious diseases consecutively included patients aged above 18 years with a confirmed diagnosis of HIV. Patients were eligible if they had been treated with ART for at least 1 year. A written informed consent was obtained from each patient before inclusion in the study.

Each participant was asked to answer a written questionnaire containing the ABQ-HIV (Additional file 1) as well as the Morisky Medication Adherence Scale (MMAS-8©), which had been translated into German. The MMAS is a validated and broadly used self-report instrument, which, containing 8 items, measures a specific medication-taking behavior. This instrument had already been used for HIV patients [15–19]. The overall MMAS score ranges from 0 to 8, with a higher score indicating better adherence. Based on the score, adherence levels can be categorized as high, medium, and low.

In addition, relevant patient characteristics and treatment-related information were collected by the study sites, using a pre-defined case report form.

The utility of the ABQ-HIV was evaluated as follows: Firstly, the properties of the separate items were assessed. This includes the examination of missing data, which provides information about the clarity of the questions. Items with a disproportionate share of missing data indicate an overstraining of the respondent regarding understanding or sensibility. Furthermore, floor and ceiling effects were assed for each item, and items with a high endorsement rate for one answer option were discussed to be excluded from the questionnaire, as these items are considered to be redundant because they add little value to the index [20, 21]. Secondly, internal reliability was evaluated by calculating Cronbach’s α; with a currently acceptable value of 0.7–0.9 [20, 22]. This also included an assessment of whether exclusion of a respective item would lead to a considerable improvement of the value of Cronbach’s α. Furthermore, split-half reliability was assessed based on the Spearman-Brown Formula, where the Spearman–Brown coefficient ≥ 0.7 was considered satisfactory [23]. Item-total correlation with an acceptable value between 0.2–0.8 [15] was also examined. Thirdly, after final decision about exclusion of items based on the evaluation of item-properties and reliability, a factor analysis with varimax rotation was used to identify potential subscales of the ABQ-HIV. Items that showed maximum loading on the same factor were considered to belong to one subscale. Fourthly, the convergent validity, which refers to the degree to which two measures that theoretically should be related are in fact related to each other, was examined in this study. Here, correlation between the self-reported adherence of each patient measured by the MMAS and the ABQ-HIV score was investigated. Furthermore, the MMAS was used to identify a meaningful threshold of the ABQ-HIV by means of receiver operating characteristic (ROC) analysis.

The study was approved by the independent Ethics Commissions of Rhineland-Palatinate (837.077.16 (10396)), Hessia (121/2016), and Saarland (132/16), Germany.

## Results

After the above outlined expert panel discussion, the drafted ABQ-HIV used for the quantitative patient survey contained 17 items. Each item was formulated as a statement. The response structure of the original ABQ was kept. This rating scale was chosen by assessing the grade of information exploitation and, on the other hand, the risk of overtaxing respondents. Finally, a 4-point Likert scale was defined, which deliberately left out a mean response option, to force the respondents to a decision. The possible answers were “strongly agree”, “generally agree”, “generally disagree” and “strongly disagree”, which were given values from 1 to 4, or rather 4 to 1, depending on the formulation of each item (a higher score indicated a higher influence of a certain barrier on a patient’s perceptions); the questionnaire given to the patients is shown in Additional file 1.

Three hundred ninety-five patients participated in the study. Twenty-five did not respond to all ABQ-HIV questions. Therefore, finally, the data of 370 patients could be included in all validation analyses. Patients who were excluded from the analysis due to missing ABQ-HIV data were on average 4.6 years older (*p* = 0.031) and more likely to be female (15.7% vs. 20.0%, *p* < .001) than those who completed the ABQ-HIV (Table 1).

**Table 1:** Baseline characteristics of patients

Variables	HIV patients who completed the ABQ-HIV	HIV patients who did not complete the ABQ-HIV	*P-*values
*N*	370	25	
Age in years – mean (SD)	51.2 (1.0)	55.8 (15.5)	0.031
Female gender – *n* (%)	58 (15.7%)	5 (20.0%)	< 0.001
Duration since first HIV diagnosis in years – mean (SD)	11.7 (0.3)	10.3 (6.1)	0.466
Duration since first HIV treatment in years – mean (SD)	8.7 (0.2)	8.6 (5.7)	0.829
Country of origin – *n* (%)			0.464
Germany	326 (88.1%)	21 (84.0%)	
Other^a^	41 (11.1%)	4 (16.0%)	
Not specified	3 (0.81%)	0 (0.0%)	
Education level – *n* (%)			0.622
No degree	24 (6.5%)	3 (12.0%)	
Apprenticeship	208 (56.2%)	17 (68.0%)	
High school degree	42 (11.4%)	2 (8.0%)	
University degree	87 (23.5%)	3 (12.0%)	
Other	2 (0.5%)	0 (0.0%)	
Not specified	4 (1.1%)	0 (0.0%)	
Employment status – *n* (%)			0.167
Employed	240 (64.9%)	14 (56.0%)	
Unemployed	45 (12.2%)	1 (4.0%)	
Pensioner/other	81 (21.9%)	10 (40.0%)	
Not specified	2 (0.5%)	0 (0.0%)	
Most common treatment regimens – *n* (%)			
Tenofovir alafenamide + emtricitabine + elvitegravir	65 (17.6%)	2 (8.0%)	
Lamivudine + abacavir + dolutegravir	58 (15.7%)	4 (16.0%)	
Tenofovir alafenamide + emtricitabine + rilpivirine	42 (11.4%)	0 (0.0%)	
Tenofovir disoproxil + emtricitabine + efavirenz	41 (11.1%)	2 (8.0%)	
Number of tablets patients needed to take per day (reported by the patient) – *n* (%)			0.906
Not specified	6 (1.6%)	2 (8.0%)	
1–3	237 (64.1%)	14 (56.0%)	
3–5	67 (18.1%)	4 (16.0%)	
5–10	60 (16.2%)	5 (20.0%)	

^a^Benin, Brazil, Bulgaria, China, Dominican Republic, France, India, Indonesia, Italy, Kameron, Kazakhstan, Kenya, Lebanon, Austria, Philippines, Poland, Portugal, Russia, Zambia, Tanzania, Thailand, Czech Republic, Turkey, Ukraine, USA

In the validation cohort of 370 patients, the mean age was 51.2 years. 15.7% were female (Table 1), the mean duration of known HIV infection was 11.7 years, and the mean time since commencement of ART was 8.7 years. Most common treatment regimens were tenofoviralafenamide/ emtricitabine/ elvitegravir (17.6%), lamivudine/ abacavir/ dolutegravir (15.7%), tenofoviralafenamide/ emtricitabine/ rilpivirine (11.4%), and tenofovirdisoproxil/ emtricitabine/ efavirenz (11.1%), which are all single-tablet regimens.

### Item properties

Most of the items show a right-skewed distribution of the scores. The only exception was item 9 (“Generally, I find it unpleasant when other people notice my medication intake.”) with a skewness of − 0.16. This item showed also the highest values for the mean (2.64) and the median (3.0).

Missing data analysis for specific items showed an outstanding value for item 11 (“Generally, I often feel bad, and sometimes I feel discouraged and depressed.”), with a missing answer in 12 cases in comparison to all other items with a maximum of three to six missing answers. However, item 11 refers to a depressed mood which may be regarded a very personal and sensitive issue for which a higher risk of non-response was expected. Therefore, we decided not to exclude this item.

In item 3 (“I trust my doctor and agree on my therapy plan together with him.”), and 4 (“My medications only help me if I take them on a strict regular basis.”), more than 90% of participants “strongly agreed”. As this suggested a ceiling effect, excluding these items was intensively discussed. Finally, we decided to keep them as they showed an acceptable item-total correlation, and since their exclusion would lead to a reduction of Cronbach’s α. Furthermore, these items seemed to be associated with a socially desirable response behavior.

### Reliability

The results of the reliability analysis are shown in Table 2. This analysis, based on all 17 initially included items, showed a Cronbach’s α of 0.708. The item-total correlations ranged from 0.120 to 0.488, whereas item 14 (“family support”) and item 16 (“consultation of the physician in case of side effects”) showed the lowest values. The assessment of Cronbach’s α after exclusion of these items confirmed that the reliability of the ABQ-HIV would be improved by removing both items. Thus, the questionnaire was reduced by eliminating item 14 and 16. Cronbach’s α of the reduced 15-item scale was 0.720. The calculation of a Spearman–Brown coefficient based on the reduced scale resulted in a split-half reliability of 0.724, which was considered to be sufficiently high to confirm the internal consistency of the ABQ-HIV.

**Table 2:** Reliability analysis

Item	17-item ABQ-HIV (Cronbach’s α: 0.708)		15-item ABQ-HIV (Cronbach’s α: 0.720)	
	Item-total correlation coefficient	Cronbach’s α if item is deleted	Item-total correlation coefficient	Cronbach’s α if item is deleted
Item 1: “I fully understand what my doctor, nurse or pharmacist has explained to me regarding my medication therapy.”	0.291	0.701	0.289	0.715
Item 2: “I can mention the names of my medicines and their scope without hesitation.”	0.302	0.695	0.308	0.708
Item 3: “I trust my doctor and agree on my therapy plan together with him.”	0.254	0.704	0.228	0.718
Item 4: “My medications only help me if I take them on a strict regular basis.”	0.207	0.705	0.193	0.719
Item 5: “Medicines are all poisonous. You should avoid taking medicines at all if possible.”	0.352	0.689	0.364	0.702
Item 6: “I feel basically healthy. Therefore, I am sometimes unsure whether I really have to take my medicines daily.”	0.273	0.699	0.286	0.712
Item 7: “I take my medicines automatically at a fixed time or on fixed occasions every day (e.g. at meal times, before going to bed).”	0.284	0.698	0.265	0.713
Item 8: “I feel that co-payments for medication are a great burden.”	0.370	0.687	0.399	0.697
Item 9: “Generally, I find it unpleasant when other people notice my medication intake.”	0.273	0.701	0.254	0.720
Item 10: “I frequently forget things on a daily basis.”	0.439	0.679	0.461	0.690
Item 11: “Generally, I often feel bad, and sometimes I feel discouraged and depressed.”	0.440	0.677	0.446	0.690
Item 12: “I frequently have problems taking my medications (e.g. swallowing, opening the package, dividing the tablets) or it is difficult for me to adhere to the accompanying conditions of the medication intake (e.g. on an empty stomach, with food or alcohol restrictions).”	0.437	0.682	0.440	0.694
Item 13: “I have difficulties adhering to my treatment plan, especially when I am away from home (e.g. at weekends, on business trips or holidays).”	0.488	0.676	0.461	0.691
Item 14: “I receive great support from my family members/friends, who I can talk to at any time and ask for help.”	0.120	0.720	–	–
Item 15: “I am really frightened about the side effects of my medicines.”	0.376	0.686	0.387	0.698
Item 16: “In case I have already noticed or in case I were to notice side effects related to my medicines: I have talked or would talk to my doctor about them as soon as possible.”	0.159	0.707	–	–
Item 17: “In case I have already noticed or in case I were to notice side effects related to my medicines: I have stopped/would stop taking my medications or took/would take less of them.”	0.189	0.706	0.199	0.720

### Factor analysis

Based on the reduced scale, factor analysis resulted in three different components of the questionnaire. The Eigenvalues of the components ranged from 1.314 to 3.313. Component 1 explained 22.1% of the variance and contained seven items (Table 3), which led to a subscale score ranging from 7 to 28. Based on the content of the items loading on component 1, this subscale represents the more unintentional barriers. Assuming that an unintentional adherence barrier exists if the item score is greater than two, 76.8% of the patients were affected by at least one unintentional barrier. The second component, which contains five items and accounted for 8.9% of the variance, can be labelled as subscale describing barriers associated with disease/treatment knowledge. The average score of this subscale (range: 5–20) was 5.9, and 10.3% of the patients reported to face at least one barrier within this subscale. Finally, item 5, item 6 and item 17, which showed a maximum loading on the third component explaining 8.8% of the variance, can be labelled as intentional adherence barriers. The score of the intentional barriers subscale ranged from 3 to 12; the average score was 5.1, and 38.1% of patients reported to be affected by at least one intentional barrier.

**Table 3:** Identified subscale by the means of factor analysis

	Component 1 “Unintentional”	Component 2 “Knowledge”	Component 3 “Intentional”
Eigenvalue	3.313	1.329	1.314
Variance explained	22.086%	8.860%	8.759%
Possible range of subscale score	7–28	5–20	3–12
Observed range of subscale score	7–27	5–11	3–12
*N* (%) of patients with at least one barrier	284 (76.8%)	38 (10.3%)	141 (38.1%)
Items			
Item 1: “I fully understand what my doctor, nurse or pharmacist has explained to me regarding my medication therapy.”		0.642	
Item 2: “I can mention the names of my medicines and their scope without hesitation.”		0.478	
Item 3: “I trust my doctor and agree on my therapy plan together with him.”		0.696	
Item 4: “My medications only help me if I take them on a strict regular basis.”		0.496	
Item 5: “Medicines are all poisonous. You should avoid taking medicines at all if possible.”			0.563
Item 6: “I feel basically healthy. Therefore, I am sometimes unsure whether I really have to take my medicines daily.”			0.749
Item 7: “I take my medicines automatically at a fixed time or on fixed occasions every day (e.g. at meal times, before going to bed).”		0.477	
Item 8: “I feel that co-payments for medication are a great burden.”	0.460		
Item 9: “Generally, I find it unpleasant when other people notice my medication intake.”	0.554		
Item 10: “I frequently forget things on a daily basis.”	0.613		
Item 11: “Generally, I often feel bad, and sometimes I feel discouraged and depressed.”	0.651		
Item 12: “I frequently have problems taking my medications (e.g. swallowing, opening the package, dividing the tablets) or it is difficult for me to adhere to the accompanying conditions of the medication intake (e.g. on an empty stomach, with food or alcohol restrictions).”	0.568		
Item 13: “I have difficulties adhering to my treatment plan, especially when I am away from home (e.g. at weekends, on business trips or holidays).”	0.468		
Item 15: “I am really frightened about the side effects of my medicines.”	0.639		
Item 17: “In case I have already noticed or in case I were to notice side effects related to my medicines: I have stopped/would stop taking my medications or took/would take less of them.”			0.693

### Convergent validity

The convergent validity was tested using the MMAS as a self-reported adherence measure. It was possible to assess the MMAS score for 367 patients. The score of the reduced 15-item ABQ-HIV scale, as well as the scores of the three subscales, correlated significantly with the MMAS score (Table 4). All correlation coefficients were negative, indicating that a higher burden of adherence barriers measured by ABQ-HIV or its subscales was associated with a lower MMAS score and thus, with a lower adherence level. Furthermore, we evaluated the average ABQ-HIV score and the respective subscale scores in patients who showed low self-reported adherence (MMAS score < 6), and compared their scores with the scores of patients with a minimum of medium adherence (MMAS score ≥ 6). Patients reporting low adherence (52 of 367; 14.2%) showed higher average scores for the ABQ-HIV and its affiliated subscales (Table 4).

**Table 4:** Convergent validity

	ABQ total score of reduced scale	Component 1 “Unintentional”	Component 2 “Knowledge”	Component 3 “Intentional”
Correlation (p-value) with the total score of MMAS^a^-items	−0.422 (p < 0.001)	−0.396 (p < 0.001)	−0.353 (*p* < 0.001)	− 0.171 (0.001)
Mean ABQ-HIV for patients …				
… with at least medium adherence (MMAS score = > 6)	24.01	13.27	5.76	4.97
…. with low adherence (MMAS score < 6)	29.60	17.00	6.84	5.65
*p*-value	< 0.001	< 0.001	< 0.001	0.053

^a^Use of the ©MMAS is protected by US copyright laws. Permission for use is required. A license agreement is available from: Donald E. Morisky, ScD, ScM, MSPH, Professor, Department of Community Health Sciences, UCLA School of Public Health, 650 Charles E. Young Drive South, Los Angeles, CA 90095–1772

**Fig. 1:**
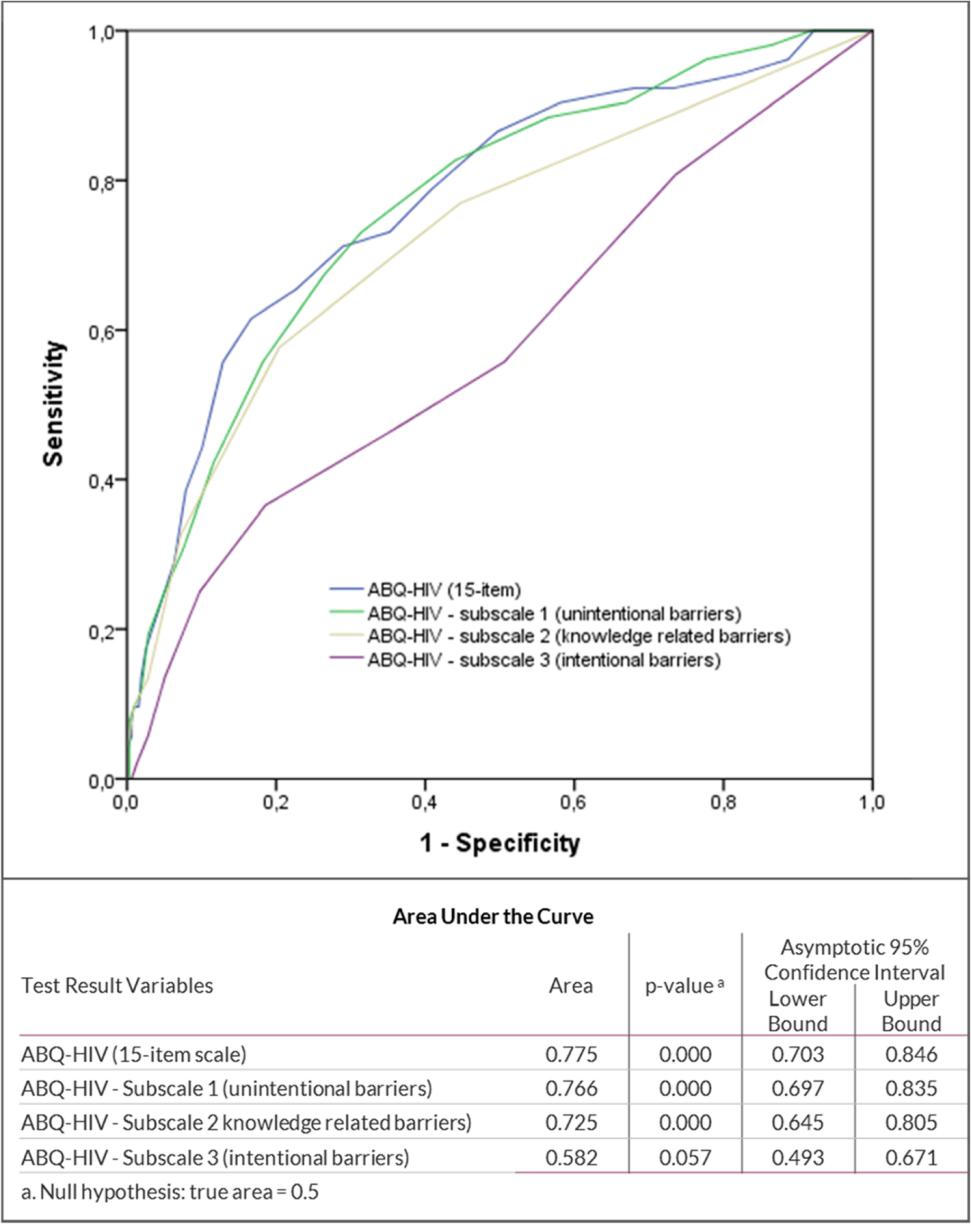
Results of the ROC analysis

The ROC curves for the overall ABQ-HIV scale and for the three subscales using the MMAS low adherence classification as state variable are shown in Fig. 1. The area under the curve (AUC) was 0.775 (*p* < 0.001) for the ABQ-HIV 15-item scale, 0.766 (p < 0.001) for subscale 1 (unintentional barriers), 0.725 for subscale 2 (knowledge related barriers), and 0.582 (p < 0.001) for subscale 3 (intentional barriers). Based on the maximum value of the sum of sensitivity and specificity, the most suitable cut-off for the ABQ-HIV scale was identified. For the overall ABQ-HIV, a cut-off of > 28 score value was identified (sensitivity: 61.5%, specificity: 83.3%). In our sample, 85 patients (23.0%) reached a score of > 28 and appeared to face a higher risk of facing substantial adherence barriers.

## Discussion

The newly developed ABQ-HIV presented here displays reasonable psychometric properties. In order to identify adherence barriers, it can reliably and easily be applied to patients with HIV. Factor analysis provided three ABQ-HIV subscales, which refer to unintentional adherence barriers, knowledge related adherence barriers, and intentional adherence barriers. All subscales demonstrated significant correlations with the used adherence self-report instrument (MMAS-8©). So, the ABQ-HIV can both be used as a tool to identify specific adherence barriers that may be present in an individual patient (use on an item-specific basis), as well as an instrument to identify certain adherence barriers clusters, which both facilitate the tailoring of adherence interventions.

No previous questionnaire focused on this issue in HIV. This validation study provided some new insights into the reasons for non-adherence in the treatment of HIV. It was observed that a high proportion of patients were affected by intentional adherence barriers. This is in contrast with other indications investigated. For example, the percentage of patients affected by intentional barriers was much lower in a sample of patients with atrial fibrillation [14]. In patients with atrial fibrillation, unintentional barriers were most common. This is also true for the investigated HIV sample, but the impact of depression/discouragement within this class of barriers was much higher than in patients with atrial fibrillation [14]. A similar impact of depression on the class of unintentional barriers could be observed in patients suffering from asthma [24]. Based on our results, a lack of information or knowledge seemed not to be a widespread problem in HIV patients.

We acknowledge some limitations to our study. This study was done with the help of a cohort of patients being taken care of by a group of infectious disease specialists in Hessia, Rhineland-Palatinate, and Saarland. Although outpatient clinics and private practices are located both in main and smaller cities, patients may neither be representative for the whole of Germany, nor for other European settings (e. g. out-of-pocket cost). So, it remains to be seen whether the ABQ-HIV shows similar properties when applied in other countries. Secondly, due to the cross-sectional design of the study, test-retest reliability and the ability to detect changes could not be assessed. Thirdly, even if the study sites were requested to include patients in a consecutive manner, there is a certain risk of a selection bias, since patients with more regular visits (and thus a better adherence behavior) were more likely to be overrepresented in the study. In addition, patients answered the questionnaire in the premises of the study site directly before or after a consultation with the treating physician. This responding environment might have increased the risk of a bias regarding socially desirable response behavior. Especially, for questionnaires containing sensitive and critical items, the compulsion to act in a socially desirable way could play a certain role. Thus, implementation of another scale like the Social Desirability Scale-17 [25] should be considered in future studies, in order to control for the tendency of respondents to answer in a manner that will be viewed favorably by health care providers (or others). Furthermore, although the ABQ-HIV was based on an existing, well-established instrument and the research team including clinical experts tried to cover all available evidence on NA causes in patients with HIV, there is a certain risk that the instrument does not cover all patient-relevant barriers. Future research is needed to fully prove the content validity of the ABQ-HIV and to assess whether the ABQ-HIV items can explain a reasonable part of existing non-adherence. Finally, we decided to use self-reported adherence to assess the convergent validity. Other measures to assess the adherence might be more powerful in validating our tool, e.g. data derived from medication event monitoring systems, but need much more resources.

In conclusion, the original ABQ could be successfully adapted for use by HIV patients. The developed ABQ-HIV is a practical, reliable, and valid instrument for identifying barriers to antiretroviral therapy adherence. The questionnaire has the potential to support physician-patient communication - knowing which kind of barriers might affect the patients would help clinicians and/or further stakeholders to approach specific issues with the patient. Although the ABQ-HIV might probably be not a measure that will be used by physician in clinical routine practice to “assess” individual patients’ adherence, it is a tool that can be applied in patient collectives to generate awareness for patients’ perspectives/barriers. Furthermore, in case adherence-promoting interventions are developed, the ABQ-HIV can be used as a tool to identify different patient clusters/segments and thus, to be able to implement interventions in a more specific / tailored way which would offer the possibility to enhance the effectiveness of adherence-supporting measures. Finally, future research is required to examine the usefulness of the ABQ-HIV in other settings and its ability to identify patient behavior changes over time.

## Additional file

Additional file 1: Adherence Barriers Questionnaire for HIV (English). (DOCX 89 kb)

## Abbreviations

ABQ: Adherence Barriers Questionnaire
ART: Antiretroviral therapy
HIV: Human immunodeficiency virus
MMAS-8©: Morisky Medication Adherence Scale
NA: Non-adherence
ROC: Receiver operating characteristic
WHO: World Health Organization
