# Supplementary material for: Adaption and validation of the adherence barriers questionnaire for HIV patients on antiretroviral therapy (ABQ-HIV)
Source: BMC Infect Dis. 2018 Nov 28;18:599. doi: 10.1186/s12879-018-3530-x (PMC6264035; doi:10.1186/s12879-018-3530-x)
Supplement: Supplementary file 1 — Adherence Barriers Questionnaire for HIV (English). (DOCX 89 kb) [file 12879_2018_3530_MOESM1_ESM.docx]

**©Adherence Barriers Questionnaire for HIV**

*Please answer each question and select to what extent the statements apply to you.*

|  |  | Strongly agree | Generally agree | Generally disagree | Strongly disagree |
| --- | --- | --- | --- | --- | --- |
| **1:** | ““I fully understand what my doctor, nurse or pharmacist has explained to me regarding my medication therapy.” |  |  |  |  |
| **2:** | “I can mention the names of my medicines and their scope without hesitation.” |  |  |  |  |
| **3:** | “I trust my doctor and agree on my therapy plan together with him.” |  |  |  |  |
| **4:** | “My medications only help me if I take them on a strict regular basis.” |  |  |  |  |
| **5:** | “Medicines are all poisonous. You should avoid taking medicines at all if possible.” |  |  |  |  |
| **6:** | “I feel basically healthy. Therefore, I am sometimes unsure whether I really have to take my medicines daily.” |  |  |  |  |
| **7:** | “I take my medicines automatically at a fixed time or on fixed occasions every day (e.g. at meal times, before going to bed).” |  |  |  |  |
| **8:** | “I feel that co-payments for medication are a great burden.” |  |  |  |  |
| **9:** | “Generally, I find it unpleasant when other people notice my medication intake.” |  |  |  |  |
| **10:** | “I frequently forget things on a daily basis.” |  |  |  |  |
| **11:** | “Generally, I often feel bad, and sometimes I feel discouraged and depressed.” |  |  |  |  |
| **12:** | “I frequently have problems taking my medications (e.g. swallowing, opening the package, dividing the tablets) or it is difficult for me to adhere to the accompanying conditions of the medication intake (e.g. on an empty stomach, with food or alcohol restrictions).” |  |  |  |  |
| **13:** | “I have difficulties adhering to my treatment plan, especially when I am away from home (e.g. at weekends, on business trips or holidays)." |  |  |  |  |
| **14:** | “I receive great support from my family members/friends, who I can talk to at any time and ask for help.” |  |  |  |  |
| **15:** | “I am really frightened about the side effects of my medicines.” |  |  |  |  |
| **16:** | “In case I have already noticed or in case I were to notice side effects related to my medicines: I have talked or would talk to my doctor about them as soon as possible.” |  |  |  |  |
| **17:** | “In case I have already noticed or in case I were to notice side effects related to my medicines: I have stopped/would stop taking my medications or took/would take less of them.” |  |  |  |  |

(ABQ-CD 180305)
